# Identifying and Avoiding tcDNA-ASO Sequence-Specific Toxicity for the Development of DMD Exon 51 Skipping Therapy

**DOI:** 10.1016/j.omtn.2019.11.020

**Published:** 2019-11-27

**Authors:** Philippine Aupy, Lucía Echevarría, Karima Relizani, Faouzi Zarrouki, Adrian Haeberli, Marek Komisarski, Thomas Tensorer, Grégory Jouvion, Fedor Svinartchouk, Luis Garcia, Aurélie Goyenvalle

**Affiliations:** 1 Université Paris-Saclay, UVSQ, Inserm, END-ICAP, 78000 Versailles, France; 2 SQY Therapeutics, 78180 Montigny le Bretonneux, France; 3 Synthena AG, CH-3012 Bern, Switzerland; 4 Sorbonne Université, INSERM, Pathophysiology of Pediatric Genetic Diseases, Assistance Publique-Hôpitaux de Paris, Hôpital Armand-Trousseau, UF Génétique Moléculaire, 75012 Paris, France; 5 Institut Pasteur, Experimental Neuropathology Unit, 75015 Paris, France; 6 LIA BAHN, Centre Scientifique de Monaco, 98000 Monaco

**Keywords:** antisense oligonucleotides, tcDNA-ASOs, sequence-specific toxicity, Duchenne muscular dystrophy, preclinical, splice switching

## Abstract

Tricyclo-DNA (tcDNA) antisense oligonucleotides (ASOs) hold promise for therapeutic splice-switching applications and the treatment of Duchenne muscular dystrophy (DMD) in particular. We have previously reported the therapeutic potential of tcDNA-ASO in mouse models of DMD, highlighting their unique pharmaceutical properties and unprecedented uptake in many tissues after systemic delivery, including the heart and central nervous system. Following these encouraging results, we developed phosphorothioate (PS)-modified tcDNA-ASOs targeting the human dystrophin exon 51 (H51). Preliminary evaluation of H51 PS-tcDNA in mice resulted in unexpected acute toxicity following intravenous administration of the selected candidate. *In vivo* and *in vitro* assays revealed complement activation, prolonged coagulation times, and platelet activation, correlating with the observed toxicity. In this study, we identify a novel PS-tcDNA sequence-specific toxicity induced by the formation of homodimer-like structures and investigate the therapeutic potential of a detoxified PS-tcDNA targeting exon 51. Modification of the H51-PS-tcDNA sequence, while maintaining target specificity through wobble pairing, abolished the observed toxicity by preventing homodimer formation. The resulting detoxified wobble-tcDNA candidate did not affect coagulation or complement pathways any longer nor activated platelets *in vitro* and was well tolerated *in vivo* in mice, confirming the possibility to detoxify specific tcDNA-ASO candidates successfully.

## Introduction

Antisense oligonucleotide (ASO)-based therapeutics have made tremendous progress in the last 20 years, and the recent approval of several drugs has increased the interest in the field even more. These successes have been made possible thanks to continuous improvement in chemistry and design of ASOs over the years. Numerous chemistries of ASO have been developed and tested in clinical settings, including 2′O-methyl- phosphorothioate (2′OMePS), 2′O-methoxyethyl-PS (2′MOE), phosphorodiamidate morpholino oligomer (PMO), as well as more constrained chemistries, such as constrained ethyl (cEt) and locked nucleic acid (LNA), offering further duplex stabilization. Whereas most of these chemistries have been well tolerated in animal models and in the clinic, some candidates have shown substantial toxicities, such as hepatotoxicity, nephrotoxicity, and thrombocytopenia.^1^ This type of acute and sequence-specific toxicity was mostly reported with modifications, such as LNA and cEt, and restricted to the specific class of gapmer-ASO, which aims at downregulating the target mRNA through the recruitment of RNase H1.^2^, ^3^, ^4^ Previous studies have actually demonstrated that these toxicities were mediated by RNase H1,^4^^,^^5^ although other mechanisms involving specific protein binding have also been suggested.^6^, ^7^, ^8^ These acute sequence-specific hepatotoxicities have never been reported for fully modified ASO, such as splice-switching ASO, employed to modulate the splicing of a target RNA.

We have previously demonstrated the potential of another constrained chemistry of ASO, the tricyclo-DNA (tcDNA), to induce exon skipping efficiently for the treatment of Duchenne muscular dystrophy (DMD)^9^^,^^10^ or induce exon inclusion for the treatment of spinal muscular atrophy (SMA)^11^. tcDNA chemistry displays interesting properties for neuromuscular disorder therapy, with particularly efficient uptake in respiratory and cardiac muscles, but also a unique ability to cross the blood brain barrier at low levels after systemic delivery.^9^, ^10^, ^11^ tcDNA conformationally constrained nucleotide deviates from natural DNA by the presence of three additional carbon atoms between C5′ and C3′, to which a cyclopropane unit is fused for further enhancement of structural rigidity.^12^^,^^13^ tcDNA-ASO can be used fully modified for splice-switching applications or as gapmers to downregulate specific targets.^14^^,^^15^ Fully modified tcDNA, containing all four tricyclo nucleobases, reveals increases in thermal stability of duplexes with complementary DNA by circa (ca) 1.2°C/mod and with complementary RNA of ca 2.4°C/mod.^12^ Systemic administration of tcDNA-ASO targeting the mouse DMD exon 23 (M23D) resulted in functional correction and neurobehavioral improvement of dystrophic mouse models.^10^ In a subsequent study, we demonstrated an encouraging safety profile for tcDNA-ASOs, which was well tolerated in mice even after high dose treatment up to 200 mg/kg/week for 12 weeks, albeit presenting typical PS-ASO accumulation features.^9^ Based on these promising results, we undertook the preclinical development of a PS-tcDNA candidate targeting the human DMD exon 51 for future clinical applications.

In this study, we report the unexpected sequence-specific toxicity of the selected tcDNA candidate, which appeared mediated by the formation of unique homodimer-like structures. We demonstrate that sequence modification allowing wobble base pairing to the target abolishes the observed toxicity without notably affecting the skipping potency. Our data show the efficacy and tolerability of repeated administration of the detoxified tcDNA candidate in the *mdx52* mouse model and therefore, validate methods to screen for toxic tcDNA candidates.

## Results

### Sequence-Specific Toxicity of H51(+67) PS-tcDNA

Previous work targeting the DMD exon 51 identified efficient ASO sequences of 20–30 nt, which led to the development of clinical candidates eteplirsen (PMO) and drisapersen (2′OMePS).^16^^,^^17^ With the consideration that tcDNA-ASO has higher RNA binding properties than PMO and 2′OMePS,^18^ their length can be significantly reduced to 15 nt without decreasing their potency.^10^ We performed an initial *in vitro* screening in human myoblasts (Figure S1) and identified the most potent 15-nt tcDNA-ASO to skip exon 51 within this region of interest. The preclinical candidate tcDNA-PS targeting region +67+81 of the DMD exon 51, named H51(+67), was therefore selected for further *in vivo* evaluation.

**Figure 1:**
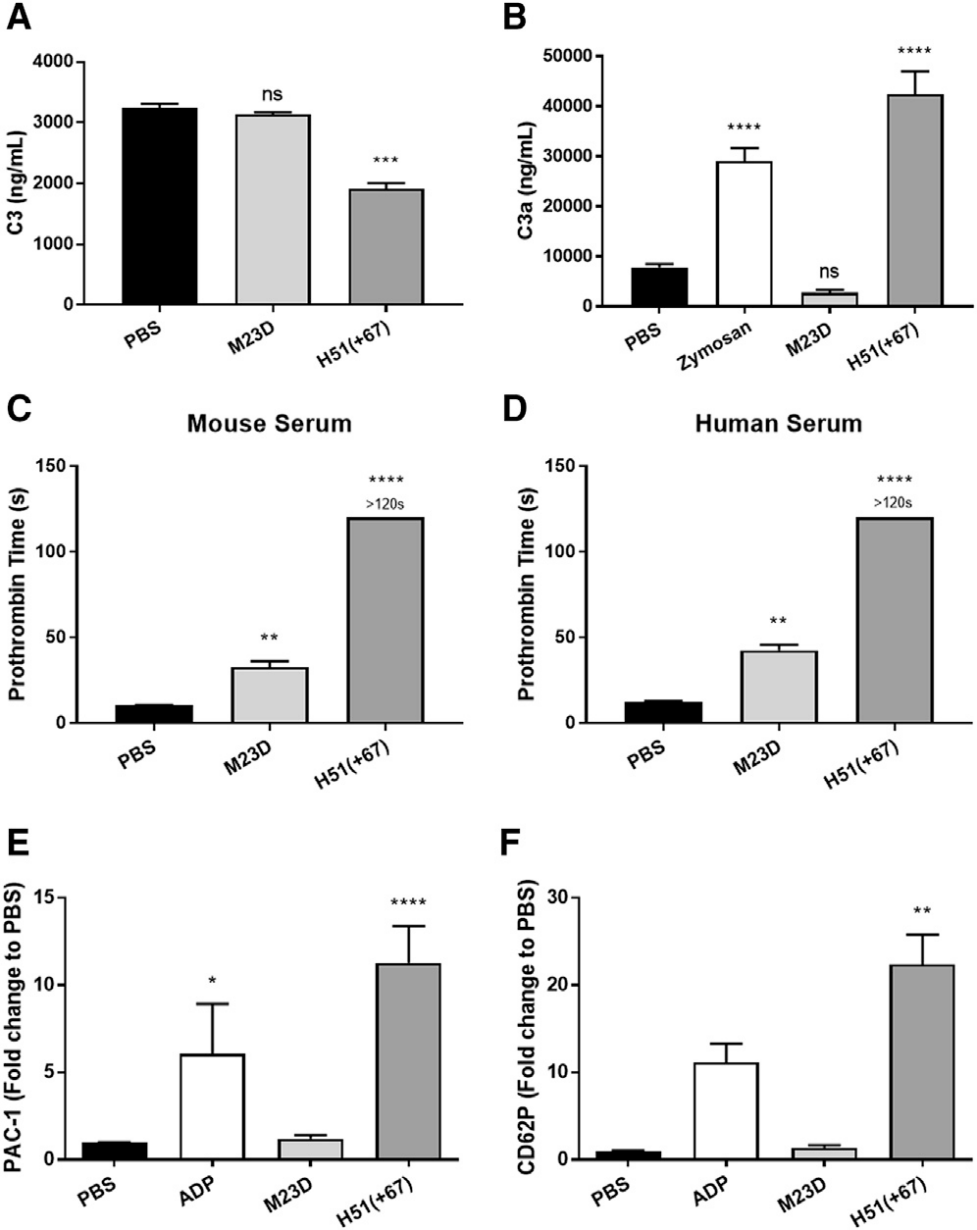
Toxic tcDNA-ASO Induces Complement Activation and Prolongation of Coagulation Times (A) Mouse serum was collected 1 h after the first injection of 200 mg/kg of tcDNA-ASO, and mouse C3 was analyzed by ELISA (PBS n = 5, M23D n = 4, H51(+67) n = 9). (B) Mouse C3a anaphylotoxin was analyzed by ELISA in mouse serum samples incubated with M23D (n = 14) or H51(+67) (n = 17). PBS (n = 25) and zymosan (n = 19) were used as negative and positive control, respectively. (C) To determine the effect on coagulation pathway, the prothrombin time (PT) was analyzed in mouse citrated plasma incubated with M23D (n = 10), H51(+67) (n = 14), or PBS (n = 19). (D) The PT was also analyzed in human citrated plasma incubated with M23D (n = 11), H51(+67) (n = 10), or PBS (n = 23). (E and F) Platelet activation was evaluated in human PRP samples incubated with PBS (negative control, n = 11), 20 μM ADP (positive control, n = 3), M23D (n = 4), or H51(+67) (n = 9), using (E) PAC1 (glycoprotein IIb/IIIa receptor) and (F) CD62P (P-selectin) markers. Values are showed as fold change compared to PBS. Results are expressed as mean ± SEM; not significant (ns) = p > 0.05, *p < 0.05, **p < 0.01, ***p < 0.001, ****p < 0.0001 compared to PBS.

Preliminary studies in C57BL/6 mice revealed unexpected and acute toxicities following intravenous (i.v.) administration of 200 mg/kg of tcDNA-H51(+67), which had never been observed with other PS-tcDNA sequences at a similar dosing regimen.^9^, ^10^, ^11^ Injected mice presented severe clinical signs, such as ventral or lateral recumbency, hypoactivity, hunched posture, piloerection, half-closed eyes, or dyspnea, a few minutes after the i.v. dosing. These effects and the hypoactivity lasted up to 3 h, after which, mice recovered and behaved normally, albeit two animals died overnight. Blood samples were collected 1 h postadministration, and complement activation was evaluated by measuring total C3 in serum. As shown in Figure 1A, we detected a significant decrease in the complement component C3 in serum from mice injected with H51(+67) as opposed to those injected with a well-tolerated tcDNA-PS targeting the M23D.^10^ The total amount of C3 decreases when C3 is cleaved to generate C3a and C3b upon complement activation. This was also confirmed *in vitro* by measuring C3a levels, which appeared significantly higher in mouse serum incubated with H51(+67), as well as with zymosan used as a positive control for complement activation (Figure 1B). These results confirmed the possibility to screen for tcDNA-mediated complement activation *in vitro*. The toxic H51(+67) also affected coagulation pathways, as revealed by the significant increase in prothrombin time (PT) in both mouse and human plasma (Figures 1C and 1D). The well-tolerated PS-tcDNA M23D slightly increased PT, which is typical of PS-ASO, but the effect of H51(+67) was significantly stronger and consistent with the observed toxicity. The activated partial thromboplastin time (aPTT) was also measured but could not discriminate between toxic and nontoxic PS-tcDNA, since aPTT is already prolonged and saturated by PS-ASO *in vitro*, as previously demonstrated.^19^ We next evaluated the ability of the different PS-tcDNA to activate platelets *in vitro* and confirmed that H51(+67) PS-tcDNA strongly activates human platelets, as demonstrated by upregulation of activated glycoprotein IIb/IIIa (PAC1; Figure 1E) and P-selectin (CD62P; Figure 1F), as opposed to the nontoxic M23D PS-tcDNA.

**Figure 2:**
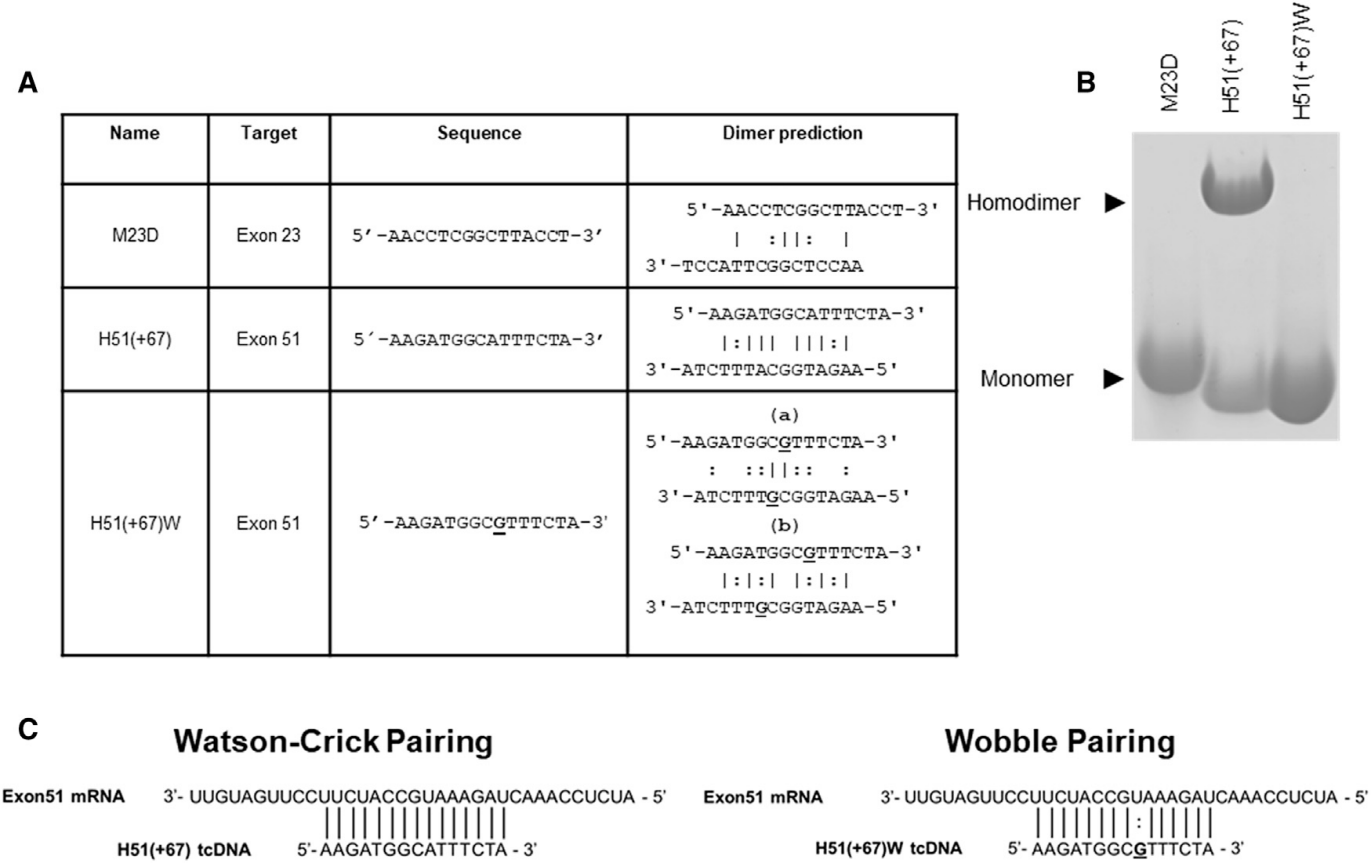
Sequence Modification Prevents Formation of Homodimer-like Structures (A) Propensity to form homodimer-like structures predicted with the OligoAnalyzer tool from IDT. The | symbol is used to depict Watson-Crick base pairing and the : symbol for wobble base pairing. For H51(+67)W, the most stable structure predicted by the OligoAnalyzer tool is shown in (Aa) and the original structure now containing wobble in (Ab). (B) Electrophoresis of tcDNA-ASOs in nondenaturing acrylamide gel to evaluate their ability to form homodimer-like structures. (C) Representation of the hybridization of tcDNA-ASO on the pre-mRNA target. H51(+67) tcDNA fully hybridizes with targeted pre-mRNA through Watson-Crick pairing (left); H51(+67)W tcDNA hybridizes with targeted pre-mRNA through one wobble pairing underlined (right).

When exploring the possible reasons for this unexpected and unique toxicity compared to many other tested sequences, we identified the propensity of the H51(+67) sequence to form homodimer-like structures. *In silico* analysis predicted higher homodimerization probabilities for the H51(+67) sequence than M23D (Figure 2A), which was confirmed by electrophoresis of the corresponding tcDNA-ASOs on nondenaturing acrylamide gels (Figure 2B).

### Detoxification and *In Vitro* Evaluation

We hypothesized that the impairment of the formation of these homodimer-like structures by modifying one nucleotide could prevent the associated toxicities. In order to maintain the hybridization to the target, we replaced an A with a G in the PS-tcDNA ASO H51(+67)W, allowing a wobble base pairing to the targeted exon 51 (Figures 2A and 2C). The resulting H51(+67)W was detected as monomers on nondenaturing acrylamide gels (Figure 2B). Measurement of the melting temperature (Tm) to the target mRNA revealed a decrease of only 3.4° from 79.6°C for H51(+67) to 76.2°C for H51(+67)W.

**Figure 3:**
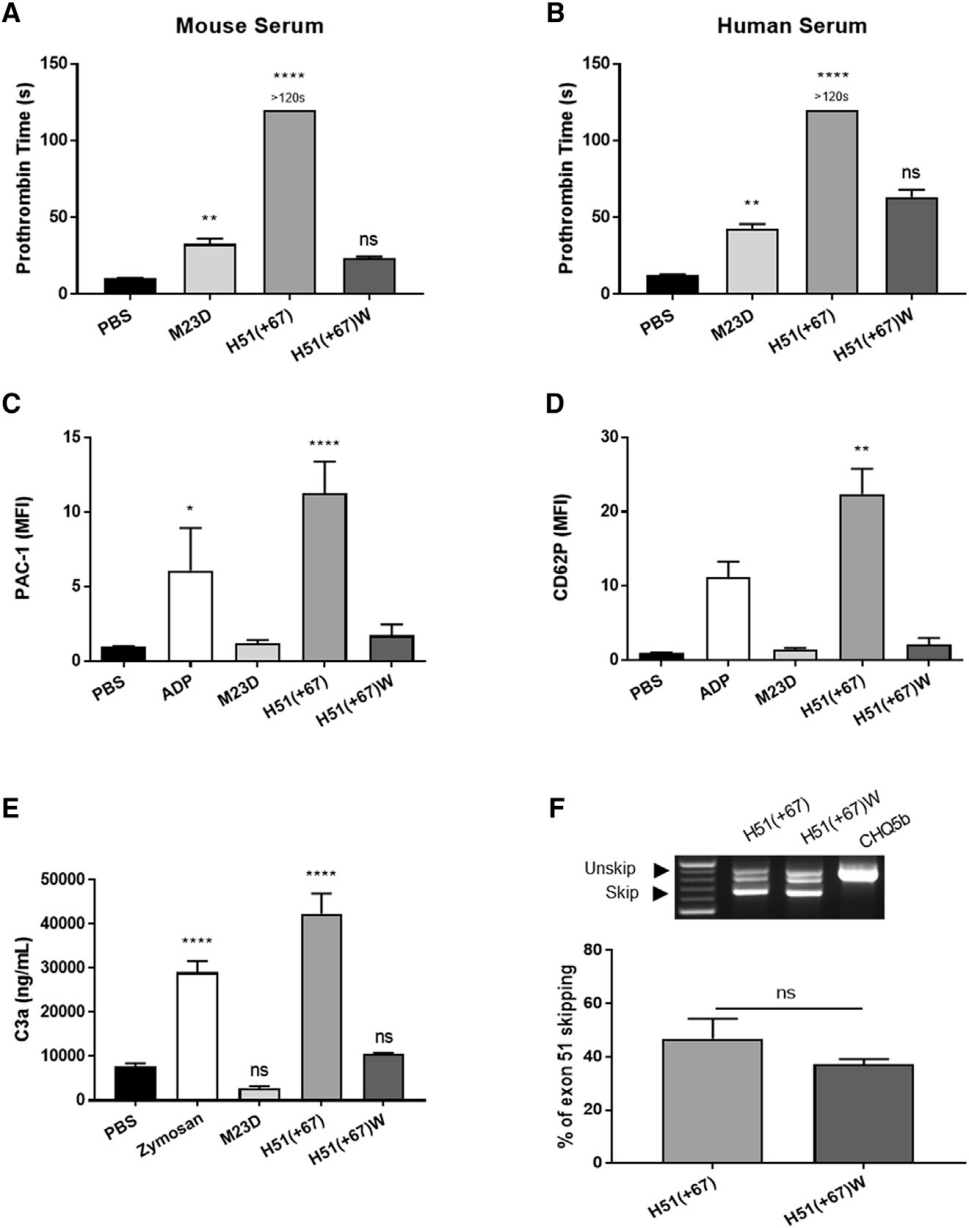
Sequence Modification Abolishes Specific Toxicity but Maintains Significant Potency *In Vitro* (A) The prothrombin time (PT) was analyzed in mouse citrated plasma incubated with M23D (n = 10), H51(+67) (n = 14), H51(+67)W (n = 5), or PBS (n = 19). (B) The PT was also analyzed in human citrated plasma incubated with M23D (n = 11), H51(+67) (n = 10), H51(+67)W (n = 2), or PBS (n = 23). (C and D) Platelet activation was evaluated in human PRP samples incubated with PBS (negative control, n = 11), 20 μM ADP (positive control, n = 3), M23D (n = 4), H51(+67) (n = 9), or H51(+67)W (n = 2) using (C) PAC1 (glycoprotein IIb/IIIa receptor) and (D) CD62P (P-selectin) markers. Values are showed as fold change compared to PBS. (E) Mouse C3a anaphylotoxin was analyzed by ELISA in mouse serum samples incubated with M23D (n = 14), H51(+67) (n = 17), or H51(+67)W (n = 3). PBS (n = 25) and zymosan (n = 19) were used as negative and positive control, respectively. (F) To determine the efficacy of H51(+67) and H51(+67)W, transfections at 300 nM in CHQ were performed (n = 3), and levels of exon 51 skipping were analyzed by nested RT-PCR (top) and qRT-PCR (bottom). Results are expressed as mean ± SEM. Not significant (ns) = p > 0.05, *p < 0.05, **p < 0.01, ****p < 0.0001 compared to PBS.

The resulting H51(+67)W PS-tcDNA did not significantly affect PT, as demonstrated in mouse and human plasma (Figures 3A and 3B), nor activate human platelets, as shown in Figures 3C and 3D, where no changes in PAC1 or CD62P levels were detected with H51(+67)W. Similarly, we measured no significant changes in the levels of C3a after incubation of mouse serum with H51(+67)W PS-tcDNA, in contrast with the toxic H51(+67) (Figure 3E), suggesting a successful detoxification.

We next verified the potency of H51(+67)W to skip exon 51 in comparison with the parent H51(+67) in human myoblasts and confirmed its ability to skip exon 51 (Figure 3F) efficiently, consistent with the minor TM to target change.

### *In Vivo* Tolerability of H51(+67)W tcDNA

**Figure 4:**
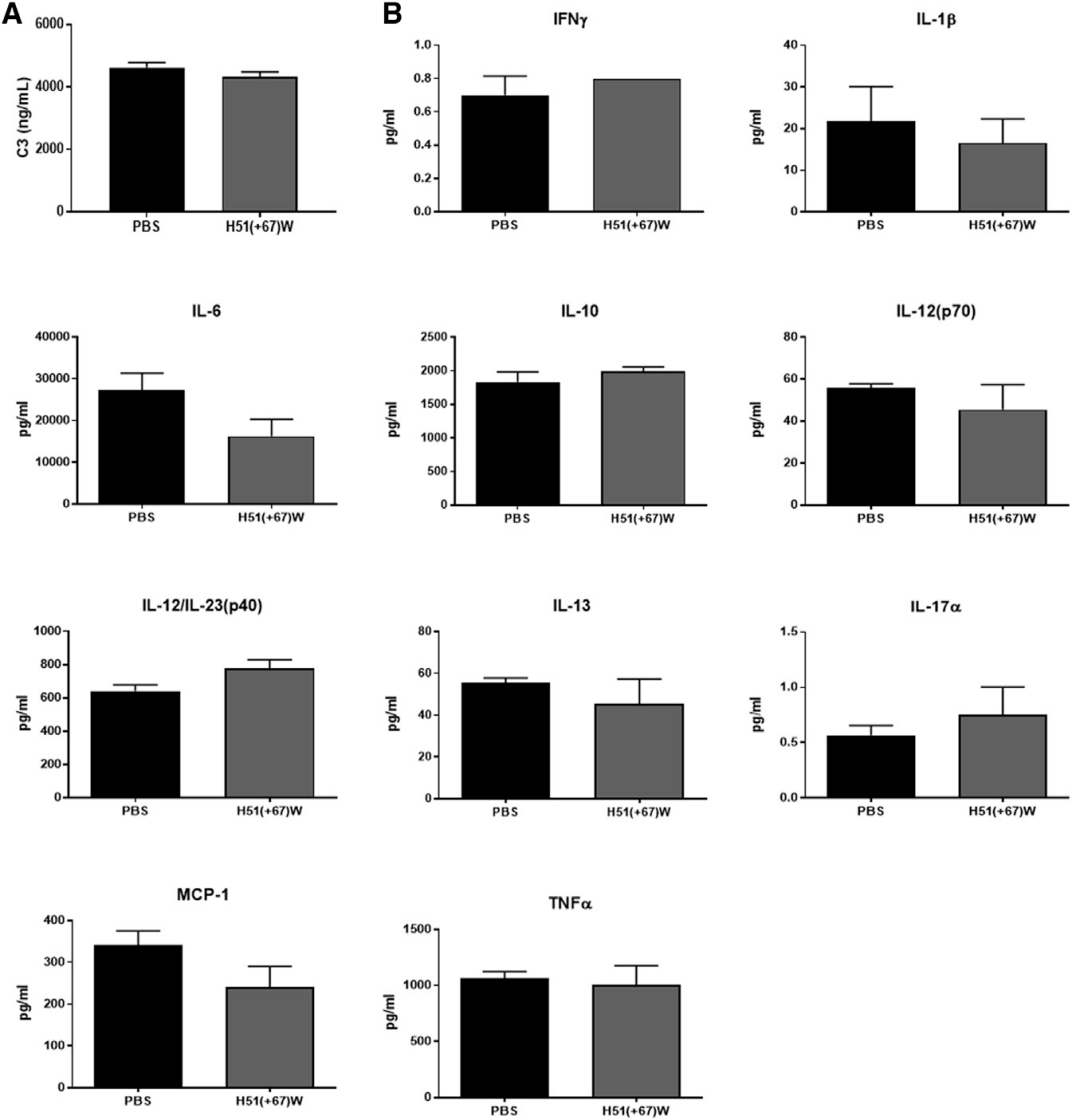
High Dose of H51(+67)W Does Not Affect Total C3 and Cytokine Levels in *mdx52* Mice (A) Mouse serum was collected 1 h after the first injection at 200 mg/kg, and mouse C3 was analyzed by ELISA (PBS n = 3, H51(+67)W (n = 4). (B) Mouse serum was collected 1 h after the first injection at 200 mg/kg, and cytokine levels were analyzed using a multiplex assay kit (PBS n = 3, H51(+67)W n = 4). Results are expressed as mean ± SEM; p > 0.05 for all groups.

We then evaluated the tolerability and efficacy of the detoxified H51(+67)W *in vivo* in mice. *mdx52* mice were injected i.v. with 200 mg/kg of H51(+67)W, and no clinical sign was reported, indicating that the detoxified compound was well tolerated. Blood samples were collected 1 h postadministration, and no significant changes in C3 levels were detected (Figure 4A), confirming the absence of complement activation also demonstrated *in vitro*. We also quantified the expression of a number of cytokines using multiplex assays and demonstrated no significant changes in interferon-γ (IFN-γ), monocyte chemoattractant protein-1 (MCP-1), interleukin (IL)-1β, IL-6, IL-10, IL-12 (p70), IL-12-p40, IL-13, IL-17α, and tumor necrosis factor-α (TNF-α) levels following i.v. dosing (Figure 4B).

### Repeated Dosing of H51(+67)W tcDNA

**Figure 5:**
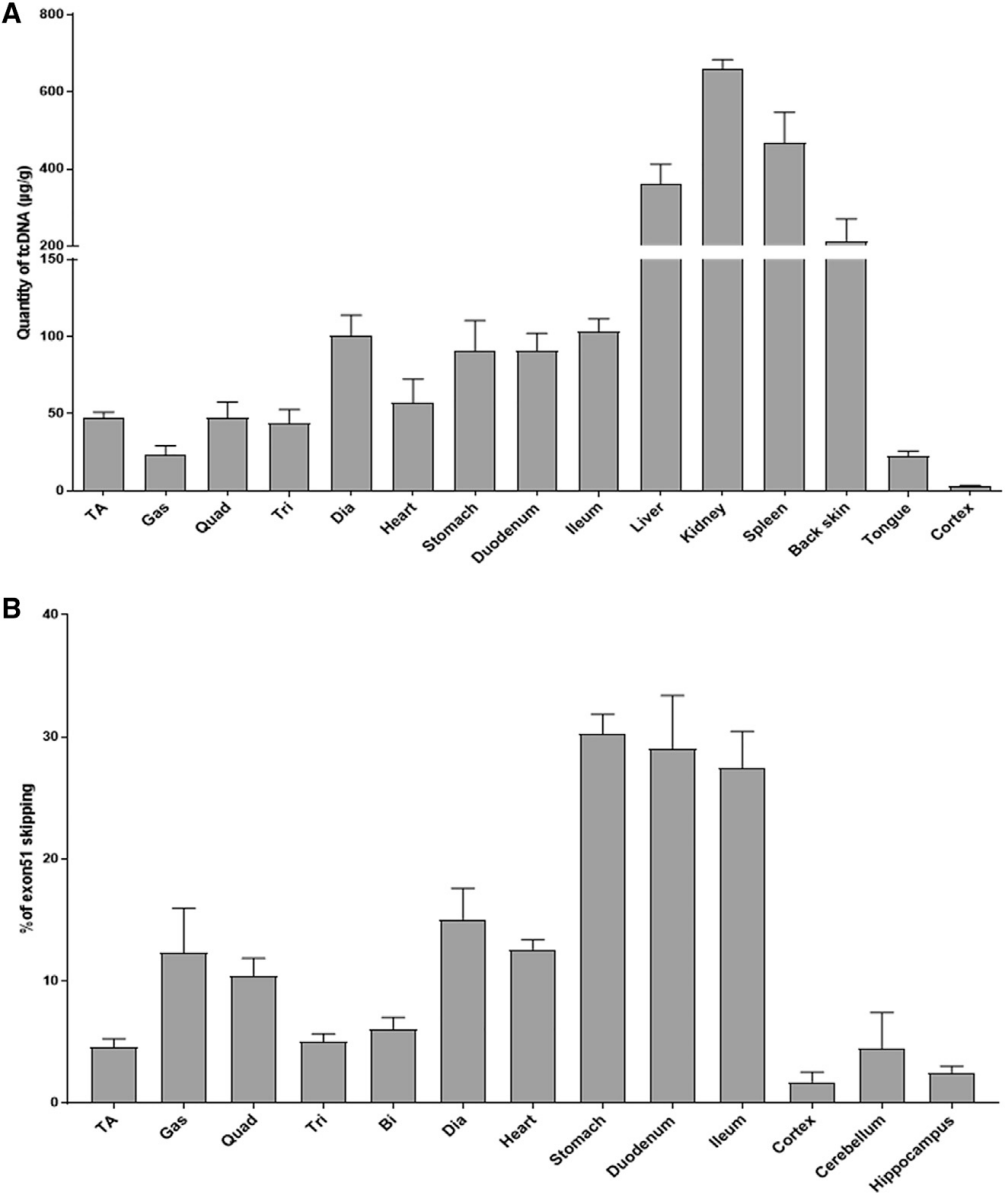
H51(+67)W Is Well Distributed to All Analyzed Tissues and Induces Exon 51 Skipping (A) Quantification of H51(+67)W tcDNA in tissues from mice treated with 200 mg/kg/week of H51(+67)W for 12 weeks and analyzed 2 weeks after the last dose. (B) Exon 51 skipping levels were evaluated by qRT-PCR in treated *mdx52* tissues, 2 weeks after the end of the 12-week treatment at 200 mg/kg/week. Results are expressed as mean ± SEM (n = 4). TA, tibialis anterior; Gas, gastrocnemius; Quad, quadriceps; Bi, biceps; Dia, diaphragm.

*mdx52* mice were treated weekly for 12 weeks, and tissues were analyzed 2 weeks after the last dose. We measured the amount of H51(+67)W tcDNA in the various tissues, and the biodistribution was similar to previously described PS-tcDNA,^9^^,^^10^ with particularly high levels in liver, kidneys, and spleen, as expected for PS-ASO (Figure 5A). Interestingly, the H51(+67)W PS-tcDNA was also detected at low levels in the cortex. qRT-PCR results revealed significant levels of exon 51 skipping in all muscle samples analyzed, with particularly high levels in smooth muscles, as well as low levels of skipping in the cortex, cerebellum, and hippocampus, confirming the previously established ability of PS-tcDNA to cross the blood brain barrier at low levels (Figure 5B and Figure S2). These data validate the potency of H51(+67)W tcDNA to skip exon 51 efficiently in *mdx52* mice, which was associated with an improvement of the respiratory function (Figure S3).

**Figure 6:**
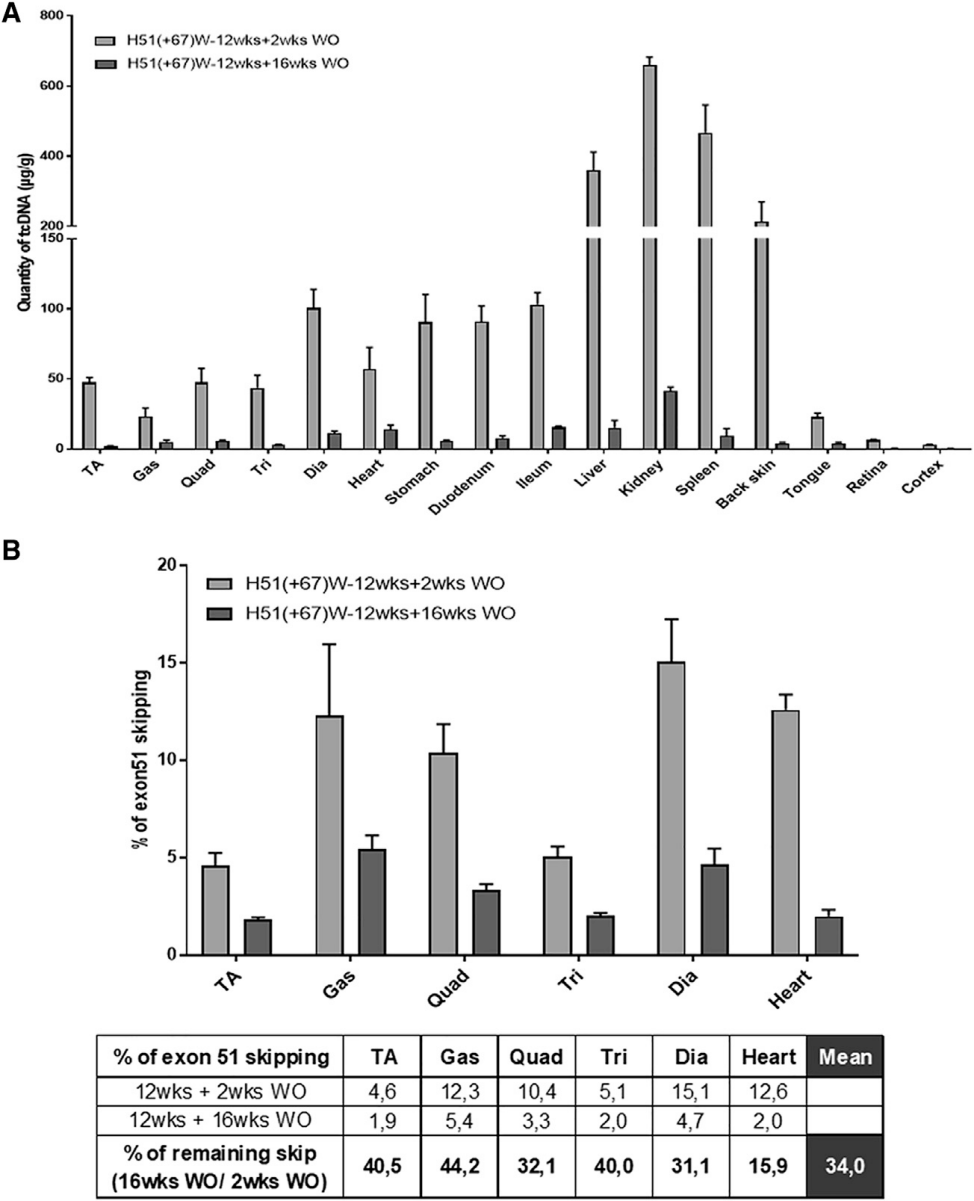
Long-Term Effect of H51(+67)W tcDNA (A) Quantification of H51(+67)W tcDNA in tissues from mice treated with 200 mg/kg/week of H51(+67)W for 12 weeks and analyzed 2 weeks and 16 weeks after the last dose. (B) Exon 51 skipping levels were evaluated by qRT-PCR in mouse tissues, 2 weeks or 16 weeks after the end of the 12-week treatment at 200 mg/kg/week. Results are expressed as mean ± SEM (n = 4 per group). Calculated percentages of the remaining levels of exon 51 skipping after a 16-week washout (WO) period compared with original levels, measured 2 weeks after the end of the treatment. TA, tibialis anterior; Gas, gastrocnemius; Quad, quadriceps; Bi, biceps; Dia, diaphragm.

In order to evaluate the longevity of the H51(+67)W PS-tcDNA effect, we also analyzed a group of treated *mdx52* mice, 16 weeks after the last injection. The 12-week, weekly treatment was well tolerated, and no clinical sign was reported during the 16-week recovery period either. We quantified the amount of tcDNA-ASO left in the various tissues and as expected, measured much less compound than 2 weeks after the last dose. Across the various muscle tissues, we detected approximately 10% of the original levels of ASO left, except in the heart, where we quantified about 25% of tcDNA left, suggesting a lower clearance rate in the cardiac muscle (Figure 6A). In contrast, only about 4% of tcDNA was found left in the high-exposure organs, such as the liver, kidney, and spleen, suggesting a higher clearance rate in these organs. More importantly, whereas only a minimal amount of tcDNA-ASO were detected in the muscle tissues, levels of exon 51 skipping were still significant, since we quantified approximately 34% of the original skipping levels across the skeletal muscles (Figure 6B and Figure S4).

### Safety Biomarkers at the End of H51(+67)W Treatment

**Figure 7:**
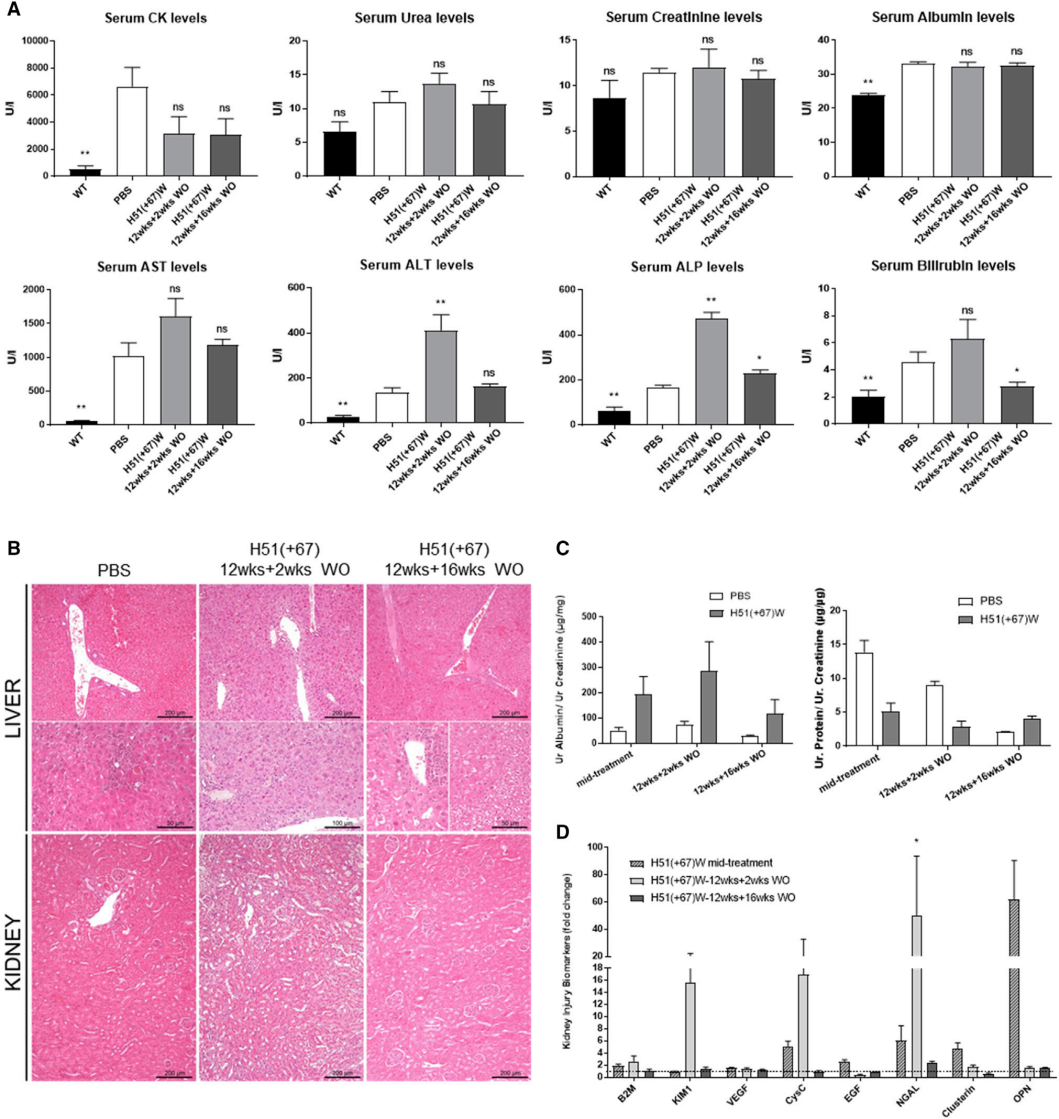
High Dose of H51(+67)W for 12 Weeks Induces Mild and Reversible Renal and Hepatic Toxicity (A) Serum biochemistry was analyzed 2 weeks (n = 4) or 16 weeks (n = 4) after the end of the 12-week treatment at 200 mg/kg/week (wild type [WT] n = 6, PBS n = 6). (B) H&E staining of liver and kidney sections from H51(+67)W-treated *mdx52* mice, analyzed 2 weeks or 16 weeks after the end of the 12-week treatment at 200 mg/kg compared to PBS-treated *mdx52* mice (PBS). Histopathological analysis revealed: (left) rare inflammatory foci in the liver of control mice with minimal anisocytosis, anisokaryosis, vacuolation, and steatosis; (middle) diffuse chronic hepatitis with multifocal necrosis of hepatocytes and almost no lesion in the kidney, 2 weeks after the end of H51(+67)W treatment; (right) rare inflammatory foci in the liver, associated with multifocal anisocytosis, anisokaryosis, and vacuolation of hepatocytes with steatosis and almost no lesion in the kidney after the 16-week recovery period. (C) Urinary albumin/creatinine (left) and protein/creatinine (right) ratios from mouse urine samples collected during treatment (H51(+67)W midtreatment, n = 4) or 2 weeks (H51(+67)W 12wks+2wks WO, n = 4) or 16 weeks (H51(+67)W 12wks+16wks WO, n = 4) after the end of the 12-week treatment. (D) KIBs were evaluated in urine samples from controls and treated mdx mice collected during treatment (H51(+67)W midtreatment, n = 4) and 2 weeks (H51(+67)W-12wks+2wks WO, n = 4) or 16 weeks (H51(+67)W-12wks+16wks WO, n = 4) after the end of the 12-week treatment tcDNA using Luminex technology. KIB levels were normalized to unit of creatinine (UCR) and expressed as fold-change ratio of their age-matched PBS controls (n = 3). Results are expressed as mean ± SEM; not significant (ns) = p > 0.05, *p < 0.05, **p < 0.01 compared to PBS.

In order to evaluate the safety of the long-term treatment of H51(+67)W, we analyzed the serum levels of various general biomarkers in mice following the 12-week treatment with 200 mg/kg/week of H51(+67)W at two different time points: 2 or 16 weeks after the last dose. The serum creatine kinase (CK) level, a marker for muscle injury, was reduced (although not statistically significant) in H51(+67)W-treated mice, 2 weeks after the end of dosing, and the level stayed low after the 16-week recovery period, which suggests the efficacy and long-term effect of the treatment to improve the dystrophic pathology of the *mdx52* mice (Figure 7A). We next quantified serum levels of urea, creatinine, and albumin, since they are first-line biomarkers of kidney function, and we did not detect any significant changes at both time points (Figure 7A). Hepatic tolerability was evaluated through measurement of bilirubin, alkaline phosphatase (ALP), and transaminases (alanine aminotransferase [ALT] and aspartate aminotransferase [AST]), and we observed a significant elevation in ALT and ALP levels, 2 weeks after the end of the treatment, which decreased again after the 16-week recovery period (Figure 7A). To investigate further potential hepatic and renal toxicities, we explored the histopathological profile of kidney and liver in H51(+67)W-treated *mdx52* mice compared with saline controls. 2 weeks after the end of the treatment, histopathological analysis of the liver revealed minimal to mild inflammatory lesions in control *mdx52* animals, consistent with their dystrophic pathology. H51(+67)W-treated mice presented diffuse chronic hepatitis with multifocal necrosis of hepatocytes (Figure 7B). In the kidney, lesions were clearly less severe. Most treated mice did not display any inflammatory lesion (only one mouse with a chronic interstitial nephritis, but one control animal also displayed small interstitial inflammatory foci; data not shown). After the 16-week recovery period, only rare inflammatory foci were still detected in the liver of both control and tcDNA-treated groups associated with some alteration of hepatocytes, such as anisocytosis, anisokaryosis, vacuolation, and steatosis (less severe for control mice). No specific lesions were detected in the kidney (Figure 7B).

With the consideration of the high accumulation of H51(+67)W in the kidney, we explored further the potential renal toxicity by measuring several kidney injury biomarkers (KIBs) in the urine of H51(+67)W-treated mice compared with saline controls. Urines were collected halfway through the 12-week treatment (i.e., after 6 i.v. injections, indicated midtreatment in Figure 7C) or at the end of the treatment after either 2 or 16 weeks of washout (WO). H51(+67)W treatment did not affect total protein level and only slightly increased levels of albumin, albeit not statistically significant (Figure 7C). Analysis of earlier KIBs revealed an upregulation of kidney injury molecule 1 (KIM-1), cystatin-C (CysC), and neutrophil gelatinase-associated lipocalin (NGAL), 2 weeks after the end of the treatment, which all returned to normal levels after the 16-week recovery period (Figure 7D). All other analyzed KIBs remained unchanged.

## Discussion

In this study, we describe for the first time an acute and unexpected toxicity induced by a fully modified PS-tcDNA ASO, which was abolished through the introduction of a wobble sequence modification. The observed toxicity was characterized by severe clinical signs occurring within 10 min of the i.v. administration of a high dose (200 mg/kg) of H51(+67) and leading to occasional acute deaths. This toxicity appeared dose dependent, since lower dosage (50 and 100 mg/kg) induced fewer and shorter clinical signs (data not shown), but also sequence dependent, since no clinical sign had ever been observed with other PS-tcDNA ASO at the same high dose.

Characterization of the observed toxicity revealed that the H51(+67) PS-tcDNA strongly activates the complement, as demonstrated by the decrease in the level of the complement component C3 following i.v. administration, as well as the increased levels of mouse C3a measured *in vitro*. The activation of complement following systemic administration of PS-ASOs is a well-characterized protein binding effect that has been extensively studied in nonhuman primates (NHPs), which are particularly sensitive to it.^20^ Henry and colleagues^21^ evidenced the direct interaction of PS-modified oligonucleotides to plasma factor H, a negative regulator of the complement cascade, which reduces free levels of the inhibitor, permitting uncontrolled amplification of the cascade. Whereas it is generally reported that complement is not activated by PS-ASOs in rodents,^22^ we observed here that toxic tcDNA sequences activate complement in mice. These results suggest that complement activation assessment can be included in rodent preclinical tcDNA screening. However, further studies are needed to establish whether the underlying mechanisms involve other complement actors/mechanisms than the factor H.

We also showed that H51(+67) impacted the extrinsic coagulation pathway by significantly increasing PT. This is actually consistent with clinical observations of the difficulties to stop the bleeding after blood sampling in mice, 1 h after the H51(+67) PS-tcDNA administration. PS-ASOs are well known to inhibit the intrinsic coagulation pathway by prolonging the aPTT,^23^ something already seen with other PS-tcDNA sequences.^19^ This effect, directly related to PS content, has been reported of little clinical significance.^24^ However, we found here that both the extrinsic and intrinsic coagulation pathways were affected by the toxic H51(+67), in which case, it could be hypothesized that additional inhibitory effects may occur and cause toxicities.

Moreover, we observed that H51(+67), but not M23D or H51(+67)W tcDNAs, activated human platelets (activated P-selectin and glycoprotein IIb/IIIa). Evidence has shown that PS ASOs without ribose modifications are able to activate platelets in a sequence-independent manner through the binding to collagen receptor glycoprotein VI (GPVI).^25^ Recently, Sewing and colleagues^26^ confirmed these findings, as well as those from Jaax et al.,^27^ who showed that PS-ASOs also interact with platelets through platelet factor 4 (PF4), a cytokine secreted by activated platelets to induce a HIT (heparin-induced thrombocytopenia)-like, immune-mediated effect. Interestingly, both mechanisms are directly related to ASO length and PS content, and they are suppressed by the addition of LNA modifications in a gapmer configuration. Our data indicate that fully modified PS-tcDNA normally do not activate platelets (as exemplified with the safe M23D sequence) and that only the toxic H51(+67) did, showing that a fully ribose-modified PS-ASO can activate platelets. It will be interesting to investigate whether platelet activation by toxic PS-tcDNA occurs through GPVII or PF4 binding, as previously reported, although our results here suggest a sequence-specific mechanism. Meanwhile, it appears relevant to screen for toxic tcDNA candidates *in vitro* by measuring activated P-selectin and glycoprotein IIb/IIIa, especially since thrombocytopenia is a known safety concern in preclinical and clinical ASO development.^24^

When exploring the possible reasons for the H51(+67) tcDNA unexpected toxicity, we identified the unique ability of this particular sequence to form homodimer-like structures, which were observed on nondenaturing gels and could not be dissolved by heating, as previously described for quadruplex.^28^ Whereas the propensity of homodimer formation can be predicted *in silico*, according to Watson-Crick base pairing, these structures are very specific of tcDNA, since we could not detect such homodimers on gels with other chemistries, such as 2′OMe, MOE, or even other constrained chemistry, such as LNA, with the same sequence (data not shown).

From these observations, we hypothesized that the impairment of homodimer formation would abolish the observed toxicity, and we therefore modified the H51(+67) by replacing an A with a G in the tcDNA sequence, allowing a wobble base pairing to the targeted exon 51. This modification only decreases the TM to target RNA from about 3°. The resulting H51(+67)W was detected as monomers on gels and did not significantly affect the extrinsic coagulation pathway, the complement, nor activate human platelets *in vitro*.

Next, we validated the potency of H51(+67)W to induce exon 51 skipping in human myoblasts, which was not significantly different from the parental H51(+67) ASO, consistent with the small TM difference calculated. i.v. Administration of H51(+67)W was well tolerated and confirmed the successful detoxification of the compound. In order to evaluate the potency of H51(+67)W *in vivo* and the tolerability of repeated dosing, we treated *mdx52* mice for 12 weeks. Analysis of the various tissues at the end of the treatment revealed a typical PS-ASO biodistribution, with particularly high levels of ASO in spleen, liver, and kidney but also significant levels of ASO in skeletal and cardiac muscles, as described for other PS-tcDNA.^9^, ^10^, ^11^ The presence of H51(+67)W in the different muscle tissues was associated with significant levels of exon 51 skipping, confirming the efficacy of the modified sequence. When we measured the amount of tcDNA-ASO left in the various tissues, 16 weeks after the last injection, we found approximately 4% of the original levels in the high-exposure organs (spleen, liver, and kidney) and 10% in the skeletal and smooth muscles. First, this indicates a higher clearance rate in high-exposure organs than in the target tissues (i.e., muscles), which is rather favorable. Second and interestingly, the levels of exon 51 skipping in muscles were approximately 34% of the original levels detected 2 weeks after the last dose, suggesting that lower amounts of ASO still induce significant exon skipping. This is likely due to a higher clearance of the unproductive ASO, which has not reached the nucleus and is therefore eliminated more rapidly than the productive part in the nucleus, still inducing exon skipping. This actually also illustrates the difficulty to determine the precise amount of ASO required in the target tissue to induce exon skipping. For example, in this study, when we calculate the amount of H51(+67)W tcDNA required to achieve 1% of exon 51 skipping (named amount for 1% efficacy [EA1%] in Table S1), 2 weeks after the last dose, we obtain an average 6.1 μg of ASO across tissues, whereas the quantity drops to 2.5 μg if we calculate this EA1% based on the 16-week WO data. This is mainly due to the limitations of ASO quantification methods, which measure the total amount of ASO in tissues as opposed to the productive part only.

Interestingly, at a similar dosage and time of analysis, our previous results, using a PS-tcDNA targeting the M23D,^10^ revealed higher levels of exon skipping, suggesting a higher potency of the M23D sequence than the H51(+67)W. This indicates that the +67+81 target sequence within the exon 51 might not be optimal, as previously suggested by Echigoya and colleagues.^29^

When evaluating the tolerability of the H51(+67)W after a 12-week treatment at high dose, we found a safety profile similar to the one previously described for other tcDNA-ASOs^9^^,^^19^ and common to most PS-ASOs.^1^^,^^30^ Overall, the treatment was well tolerated, and histopathological findings revealed a diffuse hepatitis and rare inflammatory lesions in the kidney (albeit also present in control *mdx52* mice) associated with increased levels of some serum and urinary biomarkers. These findings are in concordance with the duration and high-dose regimen applied here, but more importantly, they were reversible, and all levels went back to normal after the 16-week WO period. This safety profile appears typical for PS-ASO, and we recently demonstrated the possibility to reduce the PS content within the tcDNA backbone to decrease specifically these PS-associated features without affecting the potency of the compound.^19^

The toxicity induced by H51(+67) and described in this study appears different from the one previously reported for some LNA or cEt gapmers,^2^^,^^4^ which was essentially hepatotoxicity characterized by extensive hepatocellular necrosis and acute release of transaminases (AST and ALT) (not occurring with H51(+67); data not shown). Different mechanisms have been proposed to explain these gapmer-mediated toxicities, including (1) an off-target cleavage of many RNAs induced by RNase H1^4^^,^^5^ and (2) specific interactions with an unknown set of cellular protein.^3^^,^^6^^,^^7^ Recent work from Shen and colleagues^8^ has demonstrated that toxic PS-gapmer-containing LNA, cEt, or 2′MOE modifications were binding many cellular proteins with high avidity, altering their function, localization, and stability. This work also elegantly showed that the introduction of a single 2′OMe modification at a particular position reduced protein binding and therefore, significantly decreased hepatotoxicity and renal tubular toxicity. Interestingly, whereas we have not investigated the particular binding of the toxic H51(+67) PS-tcDNA with the cellular proteins, described by Shen and colleagues,^8^ we also observed that the introduction of a 2′OMe nucleotide in the toxic H51(+67) PS-tcDNA sequence abolished the observed toxicities (data not shown). Similar to the wobble sequence modification, the replacement of a tcDNA nucleotide by a 2′OMe nucleotide impaired the formation of homodimer-like structures. Although the precise mechanisms underlying the toxicities mediated by these tcDNA-homodimer-like structures remain to be fully elucidated, they are likely related to protein binding and PS content. We indeed observed that the equivalent H51(+67) tcDNA in a full phosphodiester backbone (which binds very poorly to protein^19^) was well tolerated, albeit also forming homodimer-like structures (data not shown).

This capacity to form homodimer-like structures also appears different from the previously reported ability of tcDNA-ASO to self-assemble into nanoparticles,^10^^,^^31^ since the phenomenon described here appears highly sequence specific as opposed to the self-assembly properties of tcDNA-ASO, which is not affected by dimer formation.

To summarize, the hereby study helps establishing further safety guidelines for the design of safe and efficient tcDNA-ASO, which could also be used for other emerging chemistries. We demonstrate in this work the feasibility of detoxifying a specific sequence by impairing tcDNA-specific homodimer formation. Our encountering unexpected and sequence-specific toxicity allowed us to develop a set of *in vitro* tests to screen for toxic candidates, which will help pave the way to the optimal tcDNA-based ASO design.

## Materials and Methods

### Antisense Oligonucleotides and Animal Experiments

Animal procedures were performed in accordance with national and European legislation, approved by the French government (Ministère de l’Enseignement Supérieur et de la Recherche, Autorisation APAFiS #6518). *mdx52* (C57BL/6)^32^ mice were obtained from Jun Tanihata and Shin’ichi Takeda from the National Center of Neurology and Psychiatry under a material transfer agreement. *mdx52* mice were bred in our animal facility at the Plateforme 2Care, UFR des Sciences de la santé, Université de Versailles-Saint Quentin, and were maintained in a standard 12-h light/dark cycle with free access to food and water. Mice were weaned at weeks 4–5 postnatal, and two to five individuals were housed per cage. All tcDNA-ASO used in this study were synthesized by Synthena (Bern, Switzerland).

For preliminary evaluation of H51(+67), ten, 6- to 8-week-old C57BL/6 mice were injected i.v. in the retro-orbital sinus with 200 mg/kg of H51(+67) PS-tcDNA-ASO under general anesthesia using 1.5%–2% isoflurane. For comparison purposes, an age-matched C57BL/6 group received an i.v. injection of 200 mg/kg of the previously described M23D-PS-tcDNA,^10^ targeting exon 23 of the DMD gene (n = 4). An additional age-matched C57BL/6 group received an equivalent volume of sterile saline as control (n = 5). 1 h after the first injection, blood samples were collected from all mice to measure complement component C3.

For long-term treatment, two groups of four, 6- to 8-week-old *mdx52* mice were injected i.v. in the retro-orbital sinus under general anesthesia using 1.5%–2% isoflurane, once a week, with 200 mg/kg of H51(+67)W PS-tcDNA for a period of 12 weeks. An age-matched *mdx52* group receiving an equivalent volume of sterile saline was included as control. 1 h after the first injection, blood samples were collected from all mice to measure complement component C3 and cytokines. Additional blood samples were collected at the end of the treatment when animals were euthanized. Urine samples were collected using metabolic cages over 24 h, directly in refrigerated tubes (4°C), between injections 6 and 7, and 2 weeks and 16 weeks after the end of the treatment. Animals were euthanized 2 weeks or 16 weeks after the end of the 12-week treatment, and muscles and tissues were harvested and snap frozen in liquid nitrogen-cooled isopentane and stored at −80°C before further analysis. The kidneys and liver were fixed in 10% neutral-buffered formalin and embedded in paraffin. Four micron-thick sections were cut and stained in hematoxylin and eosin (H&E).

Sample sizes and n values are indicated in each figure legend. Investigators were blinded for RNA and histopathology analysis.

### Serum Analysis and Urine Analysis

Analyses of mouse serum CK, ALT, AST, ALP, bilirubin, creatinine, urea, and albumin levels were performed by the Pathology Laboratory at Mary Lyon Centre, Medical Research Council, Harwell, Oxfordshire, UK. Cytokine and chemokine levels in mouse serum were analyzed by multiplex assays using (Meso Scale Discovery technology; Meso Scale Diagnostics). A U-PLEX Biomarker Group 1 was used to detect levels of IFN-γ, IL-1β, IL-6, IL-10, IL-12(p70), IL-12(p40)/IL-23, IL-13, IL-17, MCP-1- and TNF-α, according to the manufacturer’s instructions.

#### Coagulation Assays

Human plasma from healthy volunteers was obtained from the French Blood Donors Organization (Etablissement Français du Sang [EFS]). Mouse or human citrated plasma samples were incubated *in vitro* with 2 mg/mL of tcDNA for 20 min at 37°; then, PT assays were performed on a semi-automated STart Max coagulometer (Stago), following the manufacturer’s instructions.

#### Complement Activation Assays

Complement activation in mouse serum samples was measured by Microvue PS-C3 converter and SC5b-9 Plus kits (Quidel, San Diego, CA, USA). For *in vitro* complement activation studies, tcDNA was incubated with mouse serum at 37°C for 45 min. Determination of complement activation was evaluated using a mouse C3a ELISA kit (Teco Medical, Switzerland). 5 mg/mL zymosan (Complement Technology, Tyler, TX, USA) was used as positive control.

#### Platelet Activation Assays

Human citrated blood was centrifuged at 100 *g* for 15 min at room temperature, and supernatant was carefully transferred to a clean tube. An aliquot of 50 μL of platelet-rich plasma (PRP) was incubated with 2 mg/mL ASO or 20 μM ADP for 15 min at 37°C. An ADP agonist was used as positive control. Then, stimulated or control PRP was transferred to a polypropylene tube containing a mixture of CD61 (R-phycoerythrin [PE] mouse anti-human CD61; BD Biosciences; to identify resting and nonresting platelets), PAC1 (fluorescein isothiocyanate [FITC] anti-glycoprotein IIb/IIIa PAC1; BD Biosciences), and CD62P (allophycocyanin [APC] mouse anti-human CD62P; BD Biosciences), for the detection of activated platelets, and incubated 15 min in the dark. Samples were fixed with 1 mL of 1% paraformaldehyde and analyzed by flow cytometry (BD LSRFortessa; BD Biosciences).

#### Urine Analysis

Urine creatinine was measured using a Creatinine Assay Kit (R&D Systems, Minneapolis, MN), following the manufacturer’s instructions. Total protein in urine samples was measured, as previously described (PMID: 26213685). Briefly, proteins were precipitated from urine samples by adding 40 μL of water and 200 μL of prechilled acetone to 10 μL of urine. Samples were then incubated at −20°C for 30 min and then centrifuged at 14,000 *g*, 4°C, for 15 min. Pellets were resuspended in 40 μL of water, and protein concentration was measured using the Pierce BCA Assay (Thermo Scientific, Rockford, IL). Albumin from urine samples was measured using the Albumin ELISA Kit (Bethyl Laboratories, Montgomery, TX), following the manufacturer’s instructions.

Acute kidney injury (AKI) biomarker levels were analyzed by multiplex assays, using the Luminex technology. The multiplex kidney injury panels (MKI1MAG-94K, MKI2MAG-94K; Merck Millipore) were used, according to the manufacturer’s instructions, to measure levels of β-2-microglobulin (B2M), KIM-1, vascular endothelial growth factor (VEGF), CysC, epidermal growth factor (EGF), lipocalin-2-NGAL, clusterin, and osteopontin (OPN). The results were read using a Bio-Plex MAGPIX Multiplex Reader and analyzed with Bio-Plex Manager 6.1 software (Bio-Rad, France)

### Cell Transfection

The human-derived skeletal muscle cell line (CHQ) was obtained from the platform for immortalization of human cells from the Institut de Myologie. CHQ cells were grown in 40% skeletal muscle cell growth medium (Promocell, Germany), 40% F-10 (Thermo Fisher Scientific, USA) with 20% fetal bovine serum, and 1% penicillin-streptomycin (100 U/mL). 24 h before transfection, *Chq5b* cells were plated in 6-well plates (1.3 × 10^5^ cells per well). Cells were transfected with 300 nM of tcDNA and Lipofectamin LTX reagent, according to the manufacturer’s instructions (Thermo Fisher Scientific, USA). After transfection, cells were incubated for 72 h in differentiation medium (DMEM, 2% horse serum, 1% penicillin-streptomycin [100 U/mL], 1 mg/mL insulin, 1 mg/mL apotransferrin) and harvested using TRIzol reagent to isolate total RNA, according to the manufacturer’s instructions (Thermo Fisher Scientific, USA).

### RNA Analysis from Human Myoblasts

Total RNA was isolated from cultured human myoblast cells using TRIzol reagent, according to the manufacturer’s instructions (Thermo Fisher Scientific, USA). Aliquots of 500 ng of total RNA were used for RT-PCR analysis using the Access RT-PCR System (Promega, USA) in a 50-μL reaction using the external primers Ex 46Fo (5′-AGGAAGCAGATAACATTGCT-3′) and Ex 53Ro (5′-TTTCATTGAACTGTTGCCTC-3′). The cDNA synthesis was carried out at 45°C for 45 min, directly followed by the primary PCR of 30 cycles of 95°C (30 s), 55°C (1 min), and 72°C (2 min). 2 μL of these reactions was then reamplified in nested PCRs by 24 cycles of 95°C (30 s), 55°C (1 min), and 72°C (1 min) using the internal primers Ex 47Fi (5′-TTACTGGTGGAAGAGTTGCC-3′) and Ex 52Ri (5′-TGATTGTTCTAGCCTCTTGA-3′). PCR products were analyzed on 2% agarose gels. Exon 51 skipping was also measured by TaqMan qRT-PCR, as previously described, using TaqMan assays that were designed against the exon 50-51 or exon 50-53 templates using the Prime Time qPCR probe assays (Integrated DNA Technology) (Assay Ex50-51:Hs.PT.58.1681852 and assay Ex50-52: forward: 5′-CTTGGACAGAACTTACCGACT-3′; reverse: 5′-CCTCTGTTCCAAATCCTGCAT-3′; probe: 5′-/56-FAM/TCACCCACC/ZEN/ATCACCCTCTGTG/3IABkFQ/-3′. gBlocks Gene Fragments (Integrated DNA Technology) from human exon 50-52 and human exon 50-52 Delta 51 were used as standards for DNA copy number. 50 ng of cDNA was used as input per reaction, and all assays were carried out in triplicate. Assays were performed under fast cycling conditions on a Bio-Rad CFX384 Touch Real-Time PCR Detection System. For a given sample, the copy number of skipped product (exon 50-52 assay) and unskipped product (exon 50-51 assay) was determined using the standards Ex49-54 and Ex49-54 Delta51, respectively. Exon 51 skipping was then expressed as a percentage of total dystrophin.

### RNA Analysis from Mouse Muscle Sections

Total RNA was isolated from muscle sections collected during cryosection using TRIzol reagent, according to the manufacturer’s instructions (Thermo Fisher Scientific, USA). Aliquots of 500 ng of total RNA were used for RT-PCR analysis using the Access RT-PCR System (Promega, USA) in a 50-μL reaction using the external primers Ex 49Fo (5′-GATTGAAGTAACAGTTCACGG-3′) and Ex 53Ro (5′-CCAGCCATTGTGTTGAATCC-3′). The cDNA synthesis was carried out at 45°C for 45 min, directly followed by the primary PCR of 30 cycles of 95°C (30 s), 58°C (1 min), and 72°C (2 min). 2 μL of these reactions was then reamplified in nested PCRs by 30 cycles of 95°C (30 s), 58°C (1 min), and 72°C (1 min) using the internal primers Ex 50Fi (5′-TTTACTTCGGGAGCTGAGGA-3′) and the same Ex 53Ri (5′-CCAGCCATTGTGTTGAATCC-3′). PCR products were analyzed on 2% agarose gels. Exon 51 skipping was also measured by TaqMan qRT-PCR, as previously described, using TaqMan assays that were designed against the exon 50-51 or exon 50-53 templates using the Custom Assay Design Tool (Life Technologies) (Assay Ex50-51: Mm01216958_m1 and assay Ex50-53: AID1U27). An inventoried glyceraldehyde 3-phosphate dehydrogenase (GAPDH) assay was utilized as an endogenous control (Life Technologies). 50 ng of cDNA was used as input per reaction, and all assays were carried out in triplicate. Assays were performed under fast cycling conditions on a Bio-Rad CFX384 Touch Real-Time PCR Detection System, and all data were analyzed using the comparative threshold cycle (Ct) method. For a given sample, the delta-Ct values of exon 50-51 and exon 50-53 assays were used to calculate a relative abundance of unskipped and exon 51-skipped dystrophin mRNA, respectively. Exon 51 skipping was then expressed as a percentage against total dystrophin.

### Quantification of tcDNA by Infrared-Dye Hybridization Assay

Tissues were homogenized in proteinase K buffer (100 mM Tris-HCl, pH 8.5, 200 mM NaCl, 5 mM EDTA, 0.2% SDS, 2 mg/mL of proteinase K [Invitrogen]; 50 mg tissue/mL of buffer), followed by incubation overnight at 55°C in a heater block. After centrifugation at 7,000 *g* (Sorval ST8R centrifuge, 75005719 rotor) for 15 min, the supernatant was used in the assay. Lysates were incubated with a fluorescently labeled (Alexa680) probe (5′-TAGAAACGCCATCTT-3′; Eurogentec, Belgium) for 5 min at 50°C and were loaded onto a nondenaturing acrylamide-bis 15% gel with Tris-acetate buffer for 2 h at 110V. Bands were visualized and quantified using the Odyssey CLx system (Li-Cor, Germany). The quantification was performed using a standard of the H51(+67)W PS-tcDNA, diluted in a lysate from PBS-treated mouse.

### Detection of tcDNA Homodimers

20 μg of tcDNA, diluted in glycerol 50%, was loaded onto a nondenaturing acrylamide-bis 15% gel with Tris-acetate buffer for 2 h at 90V. Acrylamide gel was then incubated with Stains-All (Sigma-Aldrich) for 15 min with gentle shaking, and image was acquired with an Epson Scan.

### Respiratory Function

The respiratory function of mice was evaluated by whole-body plethysmography using an emka Technologies plethysmograph and essentially as recommended by TREAT-NMD. Briefly, unrestrained, conscious mice were placed in calibrated animal chambers, and the pressure difference between the reference and animal chambers was measured using a pressure transducer. Mice were allowed to acclimate in the chambers for 45 min at stable temperature and humidity. Data were then collected every 5 s using iox2 software (version 2.8.0.19; emka Technologies). The value of each parameter was calculated from an average of 60 recordings of 5 s, representing a total of 5 min. Inclusion criteria for each recording were more than eight respiration events by 5 s and >80% of success rate, as measured by iox software.

### Melting Temperature against Complementary RNA

tcDNA-ASO was mixed with its complementary strand at a final concentration of 2 μM per strand with 500 μL of Solution Buffer, two times (150 nM NaCl, 20 mM NaH_2_PO_4_ · 2H_2_O [pH 7.0]), qsp 1,000 μL in water. The solution was transferred in a 1-mL cuvette and placed in the UV spectrophotomer (Varian Cary Bio 100; Agilent), which is set at 6 L/min. Six drops of dimethylpolysiloxane were added on top of the aqueous solution when the samples reached the start temperature (90°C), and the following program was started 90°C > 20°C > 90°C > 20°C > 90°C > 20°C > 90°C, with a rate of 0.5°C/min and a measurement every 1°C. The melting temperatures were then determined using Cary WinUV software (Agilent).

### Statistical Analysis

Data were analyzed by GraphPad Prism7 software (San Diego, CA, USA) and shown as the mean ± the various SEM; n refers to the number of mice per group. Comparisons of statistical significance were assessed by nonparametric Mann-Whitney U tests for the comparison of two groups or Kruskal-Wallis for the comparison of three or more groups, followed by Dunn’s multiple comparison test. Significant levels were set at *p < 0.05, **p < 0.01, ***p < 0.001, and ****p < 0.0001.

## Author Contributions

P.A. and A.G. performed the *in vivo* experiments. P.A. and K.R. performed RNA and biodistribution analysis. L.E. performed the toxicology analysis. F.Z. analyzed the CNS. G.J. performed the histopathology analysis of liver and kidney. A.H., M.K., and T.T. provided the tcDNA-ASOs and performed TM analysis. F.S. characterized the homodimer-like structures on gels. A.G., P.A., and L.E. wrote the manuscript. A.G. and L.G. conceived the project, designed the experiments, and supervised the entire study.

## Conflicts of Interest

L.G. is cofounder of Synthena AG, which produces tricyclo-DNA oligomers. A.H., M.K., and T.T. are employees of Synthena AG.

## Supplemental Information

Document S1. Figures S1–S4 and Table S1

Document S2. Article plus Supplemental Information
