# Supplementary material for: Identifying and Avoiding tcDNA-ASO Sequence-Specific Toxicity for the Development of DMD Exon 51 Skipping Therapy
Source: Mol Ther Nucleic Acids. 2019 Nov 27;19:371–83. doi: 10.1016/j.omtn.2019.11.020 (PMC7063478; doi:10.1016/j.omtn.2019.11.020)
Supplement: Document S1. Figures S1–S4 and Table S1 [file mmc1.pdf]

OMTN, Volume *19*

Supplemental Information

Identifying and Avoiding tcDNA-ASO Sequence-Specific Toxicity for the Development of DMD Exon 51 Skipping Therapy

Philippine Aupy, Lucía Echevarría, Karima Relizani, Faouzi Zarrouki, Adrian Haeberli, Marek Komisarski, Thomas Tensorer, Grégory Jouvion, Fedor Svinartchouk, Luis Garcia, and Aurélie Goyenvalle

SUPPLEMENTAL MATERIAL

Figure S1

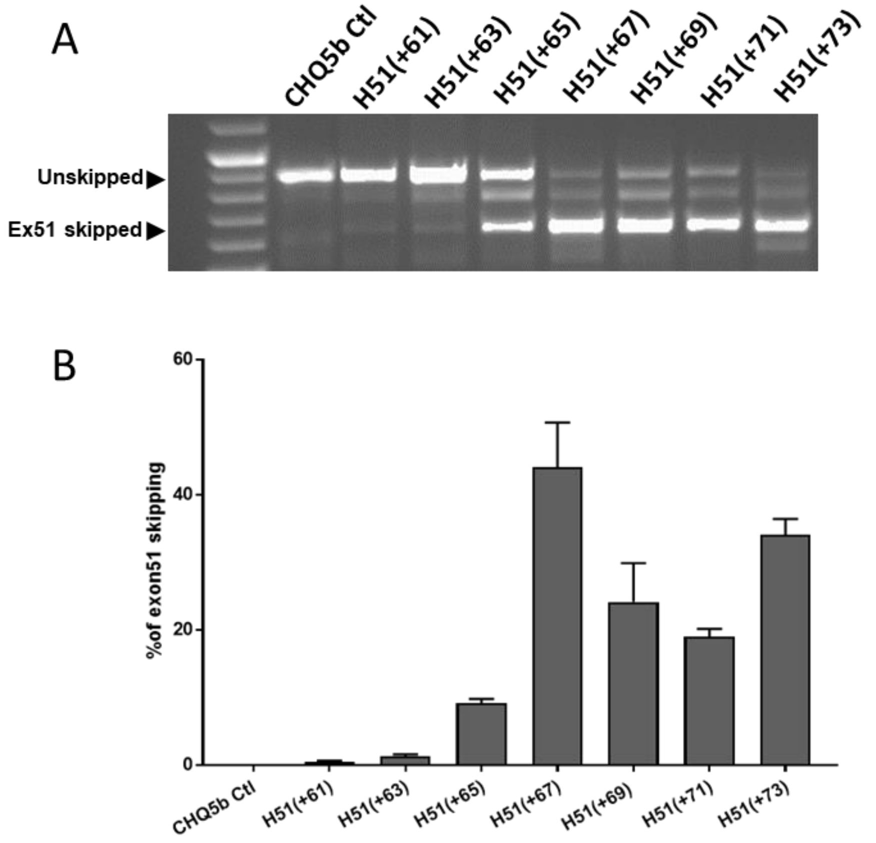

C

name	chemistry	sequence
H51(+61)	Full tcDNA-PS	5'-G*C*A*T*T*T*C*T*A*G*T*T*T*G*G-3'
H51(+63)	Full tcDNA-PS	5'-T*G*G*C*A*T*T*T*C*T*A*G*T*T*T-3'
H51(+65)	Full tcDNA-PS	5'-G*A*T*G*G*C*A*T*T*T*C*T*A*G*T-3'
H51(+67)	Full tcDNA-PS	5'-A*A*G* A*T*G* G*C*A* T*T*T* C*T*A-3'
H51(+69)	Full tcDNA-PS	5'-G*G*A*A*G*A*T*G*G*C*A*T*T*T*C-3'
H51(+71)	Full tcDNA-PS	5'-A*A*G*G*A*A*G*A*T*G*G*C*A*T*T-3'
H51(+73)	Full tcDNA-PS	5'-T*C*A*A*G*G*A*A*G*A*T*G*G*C*A-3'

Figure S1: In vitro screening of tcDNA efficacy. 300nm of the different tcDNA-ASOs were transfected in the human-derived skeletal muscle cell line CHQ5b and levels of exon 51 skipping were determined by qRT-PCR. A- representative gel of a transfection experiment. B- quantification by qRT-PCR of 3 transfection experiments. Results are expressed as Mean ± SEM. C- Sequence and chemistry of the tested compounds.

Figure S2

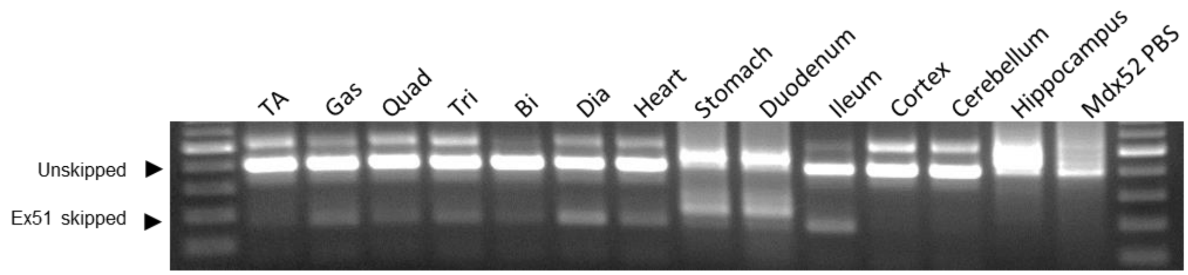

Figure S2: Evaluation of exon 51 skipping following H51(+67)W treatment. Representative gel showing exon 51 levels evaluated by RT-PCR in treated *mdx52* tissues 2 weeks after the end of the 12-week treatment at 200mg/kg/week. TA: tibialis anterior, Gas: Gastrocnemius, Quad: quadriceps, Tri: triceps, Bi: biceps, Dia: diaphragm.

Figure S3

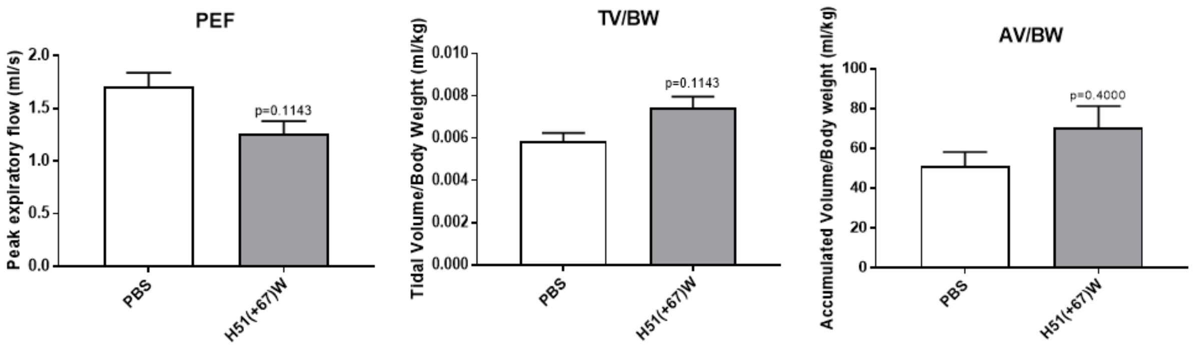

Figure S3: H51(+67)W treatment tends to improve respiratory function. Whole Body plethysmography was performed on PBS treated *mdx52* (n=3) and H51(+67)W treated *mdx52* (n=4) one week after the end of treatment. Different parameters were measured: Peak Expiratory Flow (PEF) (left), Tidal Volume normalized on Body Weight (TV/BW) (center) and Accumulated Volume Normalized on Body Weight (AV/BW) (right). Results are expressed as Mean ± SEM. P values are indicated for H51(+67)W treated *mdx52* compared to PBS.

Figure S4

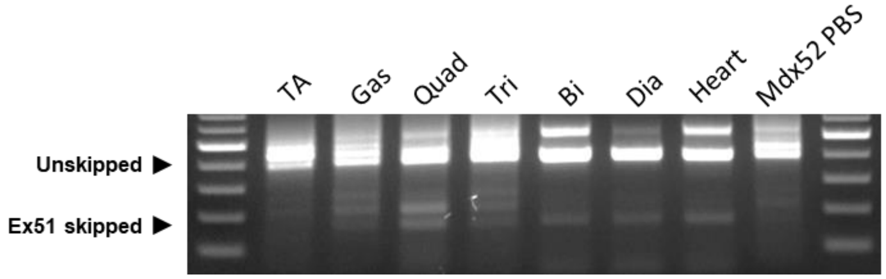

Figure S4: Evaluation of exon 51 skipping after 16 weeks of washout following H51(+67)W treatment. Representative gel showing exon 51 levels evaluated by RT-PCR in treated *mdx52* tissues 16 weeks after the end of the 12-week treatment at 200mg/kg/week. TA: tibialis anterior, Gas: Gastrocnemius, Quad: quadriceps, Tri: triceps, Bi: biceps, Dia: diaphragm.

Table S1

Amount for 1% Efficacy (1%EA)		TA	Gas	Quad	Tri	Dia	Heart	Mean
Analysis **2wks** WO	Quantity (µg/g)	47,5	23,3	47,6	43,6	100,6	57,2	
	Efficacy (%ex51 skipping)	4,6	12,3	10,4	5,1	15,1	12,6	
	**1%EA** *(Quantity/Efficacy)*	**10,3**	**1,9**	**4,6**	**8,6**	**6,7**	**4,5**	**6,1**
Analysis **16wks** WO	Quantity (µg/g)	1,7	5,2	5,9	3,3	11,3	14,2	
	Efficacy (%ex51 skipping)	1,9	5,4	3,3	2,0	4,7	2,0	
	**1%EA** *(Quantity/Efficacy)*	**0,9**	**1,0**	**1,8**	**1,6**	**2,4**	**7,1**	**2,5**

Table S1: 1%EA calculation. The 1%EA corresponds to the amount of H51(+67)W ASO required to achieve 1% of exon 51 skipping and is calculated based on the values measured after 2 weeks or 16 weeks of washout (WO).
